# Control of meiotic crossing over in plant breeding

**DOI:** 10.18699/VJGB-23-15

**Published:** 2023-04

**Authors:** S.R. Strelnikova, R.A. Komakhin

**Affiliations:** All-Russia Research Institute of Agricultural Biotechnology, Moscow, Russia; All-Russia Research Institute of Agricultural Biotechnology, Moscow, Russia

**Keywords:** meiosis, DNA, reparation, recombination, crossing over, plant breeding, мейоз, ДНК, репарация, рекомбинация, кроссинговер, селекция

## Abstract

Meiotic crossing over is the main mechanism for constructing a new allelic composition of individual chromosomes and is necessary for the proper distribution of homologous chromosomes between gametes. The parameters of meiotic crossing over that have developed in the course of evolution are determined by natural selection and do not fully suit the tasks of selective breeding research. This review summarizes the results of experimental studies aimed at increasing the frequency of crossovers and redistributing their positions along chromosomes using genetic manipulations at different stages of meiotic recombination. The consequences of inactivation and/or overexpression of the SPO11 genes, the products of which generate meiotic double-strand breaks in DNA, for the redistribution of crossover positions in the genome of various organisms are discussed. The results of studies concerning the effect of inactivation or overexpression of genes encoding RecA-like recombinases on meiotic crossing over, including those in cultivated tomato (Solanum lycopersicum L.) and its interspecific hybrids, are summarized. The consequences of inactivation of key genes of the mismatch repair system are discussed. Their suppression made it possible to significantly increase the frequency of meiotic recombination between homeologues in the interspecific hybrid yeast Saccharomyces cerevisiae × S. paradoxus and between homologues in arabidopsis plants (Arabidopsis thaliana L.). Also discussed are attempts to extrapolate these results to other plant species, in which a decrease in reproductive properties and microsatellite instability in the genome have been noted. The most significant results on the meiotic recombination frequency increase upon inactivation of the FANCM, TOP3α, RECQ4, FIGL1 crossover repressor genes and upon overexpression of the HEI10 crossover enhancer gene are separately described. In some experiments, the increase of meiotic recombination frequency by almost an order of magnitude and partial redistribution of the crossover positions along chromosomes were achieved in arabidopsis while fully preserving fecundity. Similar results have been obtained for some crops.

## Abstract (ru)

Мейотический кроссинговер является основным механизмом конструирования нового аллельного состава индивидуальных хромосом и необходим для равнозначного распределения гомологичных хромосом между гаметами. Сложившиеся в ходе эволюции параметры мейотического кроссинговера определены естественным отбором и не полностью соответствуют задачам селекционных исследований. В настоящем обзоре суммированы результаты экспериментальных работ, направленных на повышение частоты кроссоверов и перераспределение их позиций вдоль хромосом с помощью генетических манипуляций на разных этапах мейотической рекомбинации. Обсуждаются последствия инактивации и/или сверхэкспрессии генов SPO11, продукты которых генерируют мейотические двуцепочечные разрывы в ДНК, для перераспределения позиций кроссоверов в геноме различных организмов. Обобщены результаты исследований по влиянию инактивации или сверхэкспрессии генов RecA-подобных рекомбиназ на мейотический кроссинговер, в том числе у культурного томата (Solanum lycopersicum L.) и его межвидовых гибридов. Обсуждаются последствия инактивации ключевых генов системы мисмэтч-репарации. Их подавление позволило достоверно повысить частоту мейотической рекомбинации между гомеологами у межвидового гибрида дрожжей Saccharomyces cerevisiae × S. paradoxus и между гомологами у растений арабидопсиса (Arabidopsis thaliana L.). Рассматриваются попытки экстраполировать эти результаты на другие виды растений, у которых отмечены снижение репродуктивных свойств и микросателлитная нестабильность в геноме. Отдельно описаны наиболее значимые результаты по увеличению частоты мейотической рекомбинации при инактивации генов-репрессоров кроссинговера FANCM, TOP3α, RECQ4, FIGL1 и при сверхэкспрессии гена-энхансера кроссинговера HEI10. В некоторых экспериментах удалось практически на порядок повысить частоту мейотической рекомбинации и частично перераспределить позиции кроссоверов вдоль хромосом при полном сохранении плодовитости у арабидопсиса. Сходные результаты были получены для некоторых сельскохозяйственных культур.

## Introduction

Meiosis is a cell division underlying sexual reproduction, which allows species to maintain a stable set of chromosomes in a number of generations due to the proper segregation of homologous chromosomes in prophase I of meiosis. At the same time, meiosis is also a source of genetic variability, which forms due to the recombination of whole chromosomes, the exchange of regions between homologous chromosomes during crossing over, and conversion events between unpaired nucleotide bases at the site of repair of programmed doublestrand breaks (DSBs) in DNA (Mercier et al., 2015).

The entire traditional scheme of selective breeding of plant varieties and hybrids is based on the use of meiotic crossing over as the main mechanism for creating chromosomes with new combinations of alleles that are transmitted to offspring (Zhuchenko, Korol, 1985). The indispensability of meiotic crossing over for selective breeding is evident in the introgression of individual economically valuable genes from the chromosomes of wild species into the chromosomes of cultivated plants (De Muyt et al., 2009). Gaining control over the distribution of meiotic crossing over points and over the frequency of crossover exchanges will allow to construct a new allelic composition of chromosomes more effectively (Wijnker, de Jong, 2008; Lambing et al., 2017; Blary, Jenczewski, 2019).

The relevance of the study of meiotic crossing over is emphasized by many scientific reviews devoted to general issues of meiosis (Kleckner, 1996; Harrison et al., 2010; Osman et al., 2011; Crismani et al., 2013), meiotic recombination (Mézard et al., 2007; De Muyt et al., 2009; Gray, Cohen, 2016; Blary, Jenczewski, 2019; Bogdanov, Grishaeva, 2020), metabolic pathways, and crossover mechanisms (Mézard et al., 2007, 2015; De Muyt et al., 2009; Mercier et al., 2015; Gray, Cohen, 2016; Wang, Copenhaver, 2018; Bogdanov, Grishaeva, 2020), genetic control of meiotic division (Mercier et al., 2015; Gray, Cohen, 2016; Simanovsky, Bogdanov, 2018), identification and functional analysis of genes involved in meiosis (Mercier et al., 2015), epigenetic control over meiotic recombination (Yelina et al., 2015; Taagen et al., 2020), and the effect of ploidy on meiotic recombination (De Muyt et al., 2009; Lambing et al., 2017). Unlike previously published review articles, this review is devoted to practical issues of controlling the frequency and distribution of crossover exchanges between homologous chromosomes during meiosis in plants

## The role of meiotic crossing over in evolution and selection

Modern views on the molecular mechanisms of crossing over in meiosis are detailed in a number of previously published scientific literature reviews (Mercier et al., 2015; Mézard et al., 2015; Gray, Cohen, 2016; Wang, Copenhaver, 2018). The historical retrospective of the development of the theory of meiotic recombination of chromosomal DNA based on DSB repair and the experimental discovery of the “core” set of proteins: SPO11, RAD51, ZMM complex and others responsible for meiotic crossing over in most eukaryotes is presented in detail in a recent review by Yu.F. Bogdanov and T.M. Grishaeva (2020). Therefore, in this article, let us briefly note that meiotic crossing over is controlled by meiosis-specific genes, namely, meiotic recombination genes (Youds, Boulton, 2011; Bogdanov, Grishaeva, 2020). These genes are usually suppressed in somatic cells that divide by mitosis. The transition of diploid cells from division by mitosis to division by meiosis occurs as a result of acts of negative regulation, since the genes initiating meiosis turn off the genetic program of mitosis, and then the previously silent genetic program of meiosis is turned on (Turner, 2007; Bogdanov, Grishaeva, 2020).

In flowering plants, meiotic crossing over occurs in specialized cells (microsporocytes and megasporocytes) and consists of successive processes, including the creation of programmed DSBs in DNA and their repair by the mechanism of homologous recombination with the preferred use of a homologous chromosome as a template. Crossover products are crossover chromosomes that carry new combinations of allelic variants of genes (Mieulet et al., 2018).

The site of crossover exchange manifests itself in the form of a crossing between chromosomes observable under a microscope, called chiasm. In addition to crossover exchange, chiasmata also perform a structural or mechanical function: they hold chromosomes in the form of bivalents during the prophase and metaphase I of meiosis; as a result, it is the bivalents, and not individual chromosomes, that line up on the equator of the division spindle in metaphase I, which creates an opportunity for segregation of homologous chromosomes in the first division of meiosis and cell haploidization (Bogdanov, Grishaeva, 2020).

It is known that many cellular proteins required for DSB repair in the DNA of somatic cells are also involved in meiotic crossing over. However, their functions may change depending on localization, post-translational modifications, and/ or interactions with meiosis-specific proteins (Villeneuve, Hillers, 2001). This means that at some point in evolution, crossing over deviated from the function of repairing only somatic damage and acquired a function specific to meiosis. Therefore, meiotic crossing over is an evolutionary adaptation of somatic repair functions for successful sexual reproduction during the transition from the diploid to haploid phase of the life cycle. It is assumed that the evolutionarily established parameters of meiotic crossing over are regulated by natural selection to maintain the maximum adaptability of organisms to changing environmental conditions in a series of sexual generations (Zhuchenko, Korol, 1985; Wijnker, de Jong, 2008). From this point of view, the parameters of the evolutionarily established mechanism of meiotic crossing over can limit the selection process, which requires the creation of maximum genetic diversity among sexual offspring, even to the detriment of adaptability to natural habitat conditions.

The frequency of occurrence of crossovers and their distribution along chromosomes are the determining factors promoting the new and selectable genetic variability in meiosis (Zhuchenko, Korol, 1985; Wijnker, de Jong, 2008). Many studies have shown that the position of meiotic crossovers along chromosomes is non-random, strictly predetermined, non-uniform, and does not depend on genome size (Mercier et al., 2015; Mézard et al., 2015; Lambing et al., 2017; Wang, Copenhaver, 2018; Blary, Jenczewski, 2019).

According to widespread belief, there are three conceptual levels of regulation of the frequency and distribution of crossovers: obligatory crossover exchange in bivalent (crossover insurance), crossover interference, and crossover homeostasis (Simanovsky, Bogdanov, 2018; Bogdanov, Grishaeva, 2020). A possible reason for the obligatory crossover exchange in each bivalent is the mechanical function of the chiasmata (Roeder, 1997). The interference provides a nonrandom distribution of crossovers along the chromosome and their location at a greater distance from one another than is expected in case of a random distribution if there is more than one crossover per bivalent (Jones, Franklin, 2006).

Crossover homeostasis is the ability of meiotic cells to maintain the level of the number of crossovers per chromosome inherent in a given biological species, even if the number of DSBs decreases by an order of magnitude (Bogdanov, Grishaeva, 2020). In particular, in budding yeast (Saccharomyces cerevisiae (Desm.) Meyen ex E.C. Hansen), a fivefold decrease in the number of DSBs per cell was achieved, but the number of crossovers remained normal and unchanged (Martini et al., 2006). In the latter case, it is unclear whether this rule is true for all organisms. For example, in male house mice (Mus musculus), a 20–50 % decrease in the number of DSBs does not lead to a decrease in the number of crossovers and fertility (Cole et al., 2012a). However, a 60 % decrease in the number of DSBs already provokes homologue asynapsis and sterility in male mice (Kauppi et al., 2013).

Thus, in selective breeding practice, the relatively low frequency of meiotic crossovers and their determinism along chromosomes lead to the necessity for the analysis of large populations in order to identify rare recombinant genotypes that combine the desired economically valuable genes. It has also been argued that regions of chromosomes that are rarely used for crossover create additional problems for breeders, as deleterious mutations accumulate in regions of low recombination (Rodgers-Melnick et al., 2015).

## Stimulation of meiotic crossing over at the stage of creation of DNA double-strand breaks

Numerous and genetically programmed DSBs in DNA molecules are precursors of mutual genetic exchange between homologous chromosomes in prophase I of meiosis. During prophase I of meiosis, hundreds of DSBs are created along the chromosomes, generated by the evolutionarily conservative endonuclease SPO11 and some associated proteins (Keeney et al., 1997; Wang, Copenhaver, 2018).

SPO11 genes have been described in all eukaryotes whose genomes have been studied and whose protein products are similar to the A subunit of archaeal DNA topoisomerase VI (Nichols et al., 1999; Hartung, Puchta, 2001; Wu et al., 2004). In yeast, insects, and vertebrates, the SPO11 gene is represented by one copy, while the plant genome has three copies of SPO11 (Hartung, Puchta, 2001; Stacey et al., 2006). In arabidopsis (Arabidopsis thaliana L.), the SPO11-1 and SPO11-2 genes are required for meiotic recombination (Grelon et al., 2001; Stacey et al., 2006), while SPO11-3 is involved in somatic endoreplication (Hartung et al., 2002). It was previously shown that the B subunit of DNA topoisomerase VI is also required for the full formation of meiotic DSBs (Robert et al., 2016; Vrielynck et al., 2016). It forms a complex with two orthologues SPO11-1 and SPO11-2, and is absolutely necessary for the formation of the SPO11-1/SPO11-2 heterodimer in arabidopsis (Vrielynck et al., 2016).

It is assumed that the mechanism of meiotic DSB formation with the participation of SPO11 proteins is conservative, but its regulation may differ due to a set of auxiliary proteins that are less conservative in organisms from different kingdoms (De Muyt et al., 2009).

The distribution of DSBs can be considered as the first possible level of determination of future crossover formation sites. In particular, in budding yeast, from 160 to 200 DSB points are formed in each cell, and the repair of most of them leads to crossing over (Mercier et al., 2015). In other organisms, the number of DSB points significantly exceeds the number of crossing over points. For example, in arabidopsis, there are from 150 to 300 DSB points producing about 10 crossover exchanges per genome (Kurzbauer et al., 2012; Choi et al., 2013); in maize (Zea mays L.), almost 500 DSB points lead to the formation of about 20 crossover exchanges (Anderson et al., 2003). As a consequence, the site of crossover realization is selected from a wide range of potential DSB formation sites in the genome

There is an opinion that the “hot spots” of crossing over are closely associated with the “hot spots” for the formation of DSB (Wang, Copenhaver, 2018). High-resolution analysis in budding yeast shows that crossover hotspots and meiotic DSBs are concentrated in promoter-adjacent regions with low nucleosome density (Mancera et al., 2008). In humans (Homo sapiens) and house mice, “hot spots” of crossing over occur in certain DNA sequences bound by the PRDM9 protein in gene and intergenic regions not associated with transcription initiation sites (Baudat et al., 2010; Smagulova et al., 2011).

PRDM9 is a DNA-binding protein that catalyzes H3 histone (H3K4 modification) methylation, which initiates the formation of DSBs away from transcription start sites (Baudat et al., 2010; Smagulova et al., 2011). Plants do not have a PRDM9 homologue, but crossover “hot spots” do exist. “Hot spots” in arabidopsis are characterized by the fact that crossing over in them occurs up to 50 times more actively than on average for the genome (Choi et al., 2013; Yelina et al., 2015). At the same time, crossover “hot spots” could be associated with active transcription of RNA polymerase II, low nucleosome density, low DNA methylation level, as well as with intergenic regions, promoters, transcription start and stop sites, transposons, or insertion-deletion regions. The search for DNA motifs associated with crossover hotspots in arabidopsis revealed their significant enrichment in CTT, CCN, and poly A sequences (Choi et al., 2013; Wijnker et al., 2013).

Chromosomal regions associated with an increased frequency of crossing over have also been found in maize (Liu et al., 2009), wheat (Triticum aestivum L.) (Saintenac et al., 2009, 2011), and cultivated tomato (Solanum lycopersicum L.) (Demirci et al., 2017), which indicates the preservation of common mechanisms in different plant species. In most organisms, DSBs can occur along the entire length of chromosomes, however, it is surprising that 80 % of crossing over points are concentrated in about 25 % of genome regions (Blary, Jenczewski, 2019). For example, 82 % of crossovers are concentrated at the distal ends of wheat chromosome 3B, which is 19 % of its total length (Darrier et al., 2017). Therefore, despite a significant amount of species-specific information, it is currently impossible to identify a general pattern, a common or key factor in the localization of all DSBs in the genome (Bogdanov, Grishaeva, 2020). The success of practical selection can be directly related to the expansion of the range of genome regions liable to crossing over in ways including the creation of additional DSBs in regions that are rarely used to initiate DSBs or the redistribution of DSB regions

It is known that in spo11Δ-mutant budding yeast lacking their own functional SPO11 alleles, expression of the chimeric GAL4BD-SPO11 gene initiated additional DSBs at the binding site of the Gal4 protein (Peciña et al., 2002). Later, it was shown that SPO11 chimeric proteins fused with various DNA-binding protein modules (transcription factors, Cas9 nuclease, etc.) can stimulate crossing over in regions of the yeast genome with low natural recombination activity (Sarno et al., 2017). In the latter case, the authors propose their own strategy for increasing the genetic variability of gametes in plant breeding

However, it is difficult to use higher organisms with a knockout of their own SPO11 genes in selective breeding work. In arabidopsis, mutations in the SPO11-1 gene lead to a complete loss of synapsis of homologues in prophase I and their random segregation, a formation of a significant level of nonfunctional gametes, and a decrease in meiotic recombination by an order of magnitude (Grelon et al., 2001). In mice, the Spo11–/– genotype with a complete absence of DSB demonstrates chromosome asynapsis and sterility (Baudat et al., 2010). Expression of the recombinant isoform of the mouse’s own Spo11β gene made it possible to prove that the SPO11 protein level is crucial for chromosome synapsis and successful completion of meiosis (Kauppi et al., 2013). In the mei-W681 (spo11) mutants of the drosophila fly (Drosophila melanogaster), expression of the native SPO11 gene restores the wild-type phenotype (Shingu et al., 2012). Expression of the arabidopsis SPO11-1 and SPO11-2 genes or rice (Oryza sativa L.) SPO11A, SPO11B, and SPO11D genes leads to an increase in the amount of DSB in mei-W681 mutants, but this is not enough for the normal completion of meiosis (Shingu et al., 2012). The totality of the presented results shows that in higher organisms within the framework of the proposed strategy, probably, only overexpression of recombinant SPO11 genes could become a way of redistributing exchanges between homologous chromosomes.

Previously, to test this assumption, transgenic tomato plants that express the SPO11 genes from budding yeast or arabidopsis under the control of a strong constitutive 35S CaMV viral promoter were created (Komakhina et al., 2020). Using genetic analysis, it was shown that overexpression of both recombinant SPO11 genes partially disrupts the monogenic inheritance of marker alleles of the Wv:wv locus of chromosome 2 among tomato offspring. Segregation disruption at the Wv:wv locus could be the result of gene conversion due to the preferential formation of DSB in one of the Wv or wv alleles in transgenic plants. Overexpression of the SPO11 genes reduced the frequency of meiotic recombination in the region between the wv and d genes of tomato chromosome 2 by 17–18 % compared to the non-transgenic control. At the same time, a negative correlation was found between the expression level of the recombinant SPO11 genes and the frequency of recombination in the analyzed wv-d region of chromosome 2

Unfortunately, the effect of the expression of recombinant SPO11 genes on the frequency of meiotic recombination in other regions of the tomato genome remained unexplored. In general, it has been shown that the strategy of meiotic recombination induction using additional SPO11 activity, previously successfully implemented in yeast, may have limitations in plant (Komakhina et al., 2020) and insect cells (Shingu et al., 2012).

Later, a debatable opinion that DSB “hot spots” do not necessarily become crossover “hot spots” was expressed. This opinion is substantiated by the fact that a small absolute number of DSBs in “cold regions” can paradoxically turn into a relatively high frequency of realized crossing over (Bogdanov, Grishaeva, 2020).

## Stimulation of meiotic crossing over at the stage of homology search during repair of DNA double-strand breaks

During meiosis, DSBs resulting from the activity of SPO11 endonuclease are processed to 3′-single-stranded DNA ends, which then cooperatively bind RecA-like recombinases RAD51 and DMC1 (Brown, Bishop, 2014; Mercier et al., 2015). As a result, nucleoprotein filaments are formed that carry out single end invasion into the sister chromatid or the homologous chromosome (Girard et al., 2015). The 3′-singlestranded DNA ends invading the double-stranded DNA molecule are then elongated by DNA synthesis and ligation, which leads to the formation of a D-loop (displacement loop), from which a double Holliday junction is then formed (Brown, Bishop, 2014; Wang, Copenhaver, 2018).

During meiosis, DSB repair can shift towards predominant use of the homologous chromosome as a template, a process called interhomolog bias (Brown, Bishop, 2014). This process is a prerequisite for crossing over between homologous chromosomes and requires the involvement of a specific meiotic mechanism that prevents sister chromatids from being used for repair (Brown, Bishop, 2014). In particular, in arabidopsis, the meiosis-specific DMC1 protein is presumably responsible for the increased probability of DSB repair using a homologous chromosome (Kurzbauer et al., 2012).

In budding yeast and arabidopsis during meiosis, the catalytic activity of RAD51 is not necessary for the formation of interhomologous crossing over that confirms the preferential role of the DMC1 protein in this process (Cloud et al., 2012; Da Ines et al., 2013). In arabidopsis, the RAD51 protein functions within a backup pathway for DSB recovery during meiosis in the event of DMC1 dysfunction (Kurzbauer et al., 2012). In the absence of DMC1, meiotic DSBs are restored by the RAD51 protein using the sister chromatid as a template, which leads to the absence of synapsis between homologues and the appearance of univalents (Couteau et al., 1999). The presence of the DMC1 protein suppresses RAD51 activity in arabidopsis (Uanschou et al., 2013); the same is observed in meiosis in budding yeast, in which the DMC1 protein suppresses RAD51 activity (Lao et al., 2013). At the same time, in the figl1 mutants of arabidopsis, which exhibit an increased frequency of crossover exchanges, a twofold increase in the number of RAD51 foci was found in cells at the leptotene/ zygotene stages of meiosis, while the number of foci of meiosis-specific DMC1 did not change or increased insignificantly (Girard et al., 2015; Fernandes et al., 2018a). Recent results do not rule out that the role of RAD51 recombinase in meiotic crossing over may be somewhat wider than commonly believed

It is currently assumed that the choice in favor of crossing over or its absence is made during DSB processing and before the formation of the double Holliday structure (Hunter, Kleckner, 2001; Bogdanov, Grishaeva, 2020). The molecular mechanism that makes this choice continues to be discussed, but the fact of early choice is considered established (Hunter, Kleckner, 2001; Bishop, Zickler, 2004; Youds, Boulton, 2011; Gray, Cohen, 2016; Bogdanov, Grishaeva, 2020).

Structural and biochemical differences between RAD51 and DMC1 proteins are not very large (Sheridan et al., 2008). However, a large number of protein factors have been found that are required for proper loading, stabilization, and/or activation of these eukaryotic recombinases (Mercier et al., 2015).

It is known that bacterial recombinase RecA has 40 to 60 % homology with eukaryotic recombinases, but unlike them, it is universal and capable of different and even unique functions without the participation of helper proteins and with greater efficiency (Baumann, West, 1998; Lanzov, 2007). It was shown that the expression of the recA gene from Escherichia coli triples the number of DSBs restored by the mechanism of homologous recombination and more than doubles the number of sister chromatid exchanges in the somatic cells of tobacco plants (Nicotiana tabacum L.) (Reiss et al., 1996, 2000). This suggested that the expression of the recA gene in plant cells in prophase I of meiosis can also change the number and distribution of crossover exchanges between homologous chromosomes (Komakhin et al., 2010). It was later shown that the expression of the recA gene from E. coli under the control of a strong and constitutive CaMV35S promoter in cultivated tomato leads to an increase in the frequency of meiotic recombination between the wv and d genes of chromosome 2 by 50 % compared with the non-transgenic control (Komakhin et al., 2012).

The molecular mechanism that allowed to increase the frequency of meiotic recombination in the transgenic tomato remained unclear at the time these results were published. Later, it became known that the yeast Top3 topoisomerase negatively affects meiotic crossing over since it specifically destroys the D-loops formed by the yeast Rad51/Rad54 proteins (Fasching et al., 2015). However, D-loops formed by the bacterial RecA protein proved to be resistant to destruction by the Top3 protein. It has also been found that arabidopsis plants carrying top3α mutant alleles show a 1.5 to 2.5-fold increase in meiotic recombination frequency (Séguéla-Arnaud et al., 2015). Probably, in transgenic tomato plants expressing the recA gene, an increase in the frequency of meiotic recombination could be due to the formation of D-loops by the bacterial RecA protein, which could not be destroyed by the tomato TOP3α protein, resulting in an increase of the recombination frequency.

An attempt to apply this experimental approach to increase crossover exchanges between chromosomes of different tomato species showed an ambiguous result (Komakhin et al., 2019). In particular, none of the three combinations of crossing a cultivated tomato expressing the recA gene and wild tomato species S. cheesmaniae, S. pimpinellifolium, and S. habrochaites showed a significant increase in the frequency of recombination between the marker genes of chromosome 2. It is assumed that the factor limiting recombination between chromosomes from different species is the mismatch repair system, which eliminates mismatched bases in DNA at the DSB repair site (Chambers et al., 1996; Emmanuel et al., 2006; Strelnikova et al., 2021). This assumption is based on the fact that in interspecific tomato hybrids, due to the increased level of DNA polymorphism between chromosomes of different species, one should expect a more active resistance of mismatch repair to meiotic crossing over than in interline hybrids of cultivated tomato.

## Stimulation of meiotic crossing over at the stage of correction of unpaired bases at the site of DNA double-strand breaks reparation

During meiotic crossing over between homologous chromosomes, regions of heteroduplex DNA containing unpaired bases can arise locally. The mismatch repair system eliminates these regions.

The mismatch repair system is a highly conservative way of maintaining DNA integrity that exists in all organisms. The first step of this pathway in eukaryotes, mismatch recognition, is performed by homologues of prokaryotic MutS proteins, viz. MSH proteins. Eight of them were described in eukaryotes, from MSH1 to MSH8. MSH7 is found only in plants (Culligan, Hays, 2000), while MSH8 is found in the phylum Euglenozoa (Sachadyn, 2010). MSH proteins recognize unpaired bases as heterodimers. The heterodimer designated MutSa (MSH2-MSH6) repairs mismatches or 1–2 nucleotide loops (Acharya et al., 1996; Genschel et al., 1998). The MutSb heterodimer (MSH2-MSH3) recognizes larger loops containing up to 14 nucleotides (Modrich, 1991; Marti et al., 2002). Plants form an additional heterodimeric complex known as MutSc (MSH2-MSH7) (Culligan, Hays, 2000), which is involved in meiotic recombination (Lloyd et al., 2007). In meiosis, the mismatch repair system is able to destroy heteroduplex DNA and suppress crossing over (Cole et al., 2012b).

Inactivation of the MSH2 gene in interspecific yeast hybrids S. cerevisiae × S. paradoxus increases the recombination frequency between homeologous chromosomes up to 5.5 times and also increases the viability of spores (Hunter et al., 1996). In arabidopsis plants, knockout of the MSH2 gene (mutation msh2-1) increases microsatellite instability and somatic recombination, which indicates a decrease in the efficiency of the mismatch repair system in plant cells (Leonard et al., 2003). In another study, it was shown that the msh2-1 mutation increased by 40 % the frequency of meiotic recombination between marker genes of fluorescent proteins in an isogenic background of arabidopsis (Landsberg erecta ecotype) (Emmanuel et al., 2006).

These results allowed to apply the strategy of suppressing mismatch repair by inhibiting the expression of the MSH2 and MSH7 genes to increase the frequency of crossover exchanges in other plant species. In particular, in cultivated tomato, the inhibition of the expression of the MSH2 and MSH7 genes was performed by three independent scientific groups at different times either using RNA interference (RNAi) (Tam et al., 2011; Sarma et al., 2018; Strelnikova et al., 2021) or using a dominant-negative construct with the mutant MSH2-DN2 protein gene from arabidopsis (Tam et al., 2011).

The use of a dominant-negative construct or inhibition of the MSH7 transcript by RNAi allowed a non-substantial increase by 17.8 % in the frequency of meiotic recombination between homeologues in a cultivated tomato heterozygous by chromosome 8 from S. lycopersicoides Dunal (Tam et al., 2011). At the same time, silencing of the MSH2 gene transcript with RNAi delivered pronounced negative consequences for the fertility of tomato plants (Sarma et al., 2018; Strelnikova et al., 2021), especially when using the strong pro-SmAMP2 plant promoter to control the expression of the RNAi construct (Strelnikova et al., 2021). In recent experiments, it was convincingly shown that the highly effective RNAi of the MSH2 gene leads to phenotypic anomalies in cultivated tomato plants: growth and flowering retardation and formation of a reduced number of seeds (Sarma et al., 2018; Strelnikova et al., 2021). In cases where the RNAi of the MSH2 gene was moderate, tomato plants were fertile, but no increase in the frequency of meiotic recombination was found (Tam et al., 2011; Strelnikova et al., 2021).

These results show that in tomato plants, in contrast to arabidopsis plants, suppression of the MSH2 gene by RNAi to increase the frequency of meiotic recombination has significant limitations. Probably, there is a certain level of expression of the MSH2 gene, which is critical for the viability of tomato plants. This may be due to the fact that, in contrast to arabidopsis plants, the MSH2 gene in tomato performs an additional cellular function necessary for plant fertility. This may be the reason why spontaneous or induced msh2 mutants have not yet been described among various tomato species.

It should be noted that the repression of mismatch repair has a negative effect on the stability of the genome and the reproductive properties of many other plant species besides tomato. It was shown that a knockout mutation of the MSH2 gene in arabidopsis plants after several generations led to an intensive accumulation of various mutations in the genome, a partial loss of fertility, and a decrease in the number of seeds (Leonard et al., 2003; Hoffman et al., 2004). Another study showed that a mutation in the MLH1 gene, which is also a part of the mismatch repair system, leads to reproductive defects in arabidopsis plants (Dion et al., 2007).

Inhibition of MSH2 gene expression using two different strategies leads to numerous phenotypic anomalies and microsatellite instability in somatic potato hybrids (Rakosy-Tican et al., 2019). RNAi of the MSH7 gene in transgenic barley plants (Hordeum vulgare L.) leads to a decrease in the number of seeds and pollen viability (Lloyd et al., 2007). In wheat plants, the msh7-3D mutation also reduces pollen viability but does not affect plant fertility (Serra et al., 2018). Overall, these results confirm that the strategy of stimulating meiotic recombination by suppressing mismatch repair in different plant species can lead to impaired reproductive functions.

## Stimulation of meiotic recombination at the stage of D-loop resolution

As already mentioned in the section “Stimulation of meiotic crossing over at the stage of creation of DNA doublestrand breaks”, in most eukaryotes, the number of DSBs significantly exceeds the number of crossovers. This suggests that there are negatively acting metabolic mechanisms that prevent the resolution of part of the DSBs through the crossover pathway

The choice of the DSB repair mechanism in favor of a crossover or non-crossover pathway occurs at the early stages of DSB repair, when a single-stranded DNA-protein filament invades a homologous DNA molecule and causes the formation of a D-loop in it (Hunter, Kleckner, 2001; Bishop, Zickler, 2004; Bogdanov, Grishaeva, 2020). D-loops that arise during the homology search step using RAD51 and DMC1 recombinases can be transformed via various metabolic path-ways, leading either to crossovers between homologous chromosomes or to non-reciprocal exchange (without crossing over) between them

Currently, two ways of crossing over implementation, leading to the appearance of either class I or class II crossovers, are most fully described (Gray, Cohen, 2016). Class I crossovers are products of the activity of a group of proteins collectively referred to as ZMM (Zip1, Zip2, Zip3, Zip4, Msh4 and Msh5, Mer3) that stabilize intermediate D-loops, promoting the formation of a double Holliday structure (Hunter, 2015). The MLH1 and MLH3 proteins in combination with EXO1 promote the transformation of the Holliday structure into class I crossovers (Ranjha et al., 2014).

Class I crossovers are not randomly distributed along chromosomes, as they reduce the likelihood of adjacent crossovers in close proximity of them (Wang et al., 2015). This phenomenon is commonly referred to as interference. In addition, the D-loops (as a recombination intermediate) can be converted by structure-specific endonucleases, including the MUS81 enzyme, producing class II crossovers that are not subject to interference (Berchowitz et al., 2007; Wang, Copenhaver, 2018; Bogdanov, Grishaeva, 2020). There are known double mutants of arabidopsis at both msh4 and mus81 genes which control crossing over pathways I and II, respectively; despite this, these mutants show a residual 5–10 % of crossovers (Higgins et al., 2008). However, the mechanism that generates these residual crossovers is unclear; possibly, it is active only when the main crossover pathways I and II are disrupted (Osman et al., 2011; Mercier et al., 2015; Lambing et al., 2017)

There are organisms in which only one of the two major pathways of crossover formation is present. In particular, in fission yeast (Schizosaccharomyces pombe Lindner) and mold (Aspergillus nidulans P. Michel ex Haller) only pathway II is present, which is not susceptible to interference. In contrast, in the soil nematode (Caenorhabditis elegans Dougherty), only interfering pathway I is known. In plants, both crossing over pathways were found, but in different proportions. For example, in arabidopsis and tomato, class I crossovers amount to 70 to 90 %, and the rest belong to class II (Lhuissier et al., 2007; Higgins et al., 2008; Macaisne et al., 2011; Anderson et al., 2014).

Recently, using molecular genetic studies, arabidopsis was found to contain protein factors that act against the conversion of DSBs into class II crossovers: DNA helicase FANCM (Fanconi anemia complementation group M) (Crismani et al., 2012; Girard et al., 2014), FIGL1 (AAA-ATPase FIDGETINLIKE1) (Fasching et al., 2015; Girard et al., 2015), BTR complex of DNA helicases RECQ4A and RECQ4B and topoisomerase TOP3α (Séguéla-Arnaud et al., 2015).

Previously, the At1g35530 gene was found in the arabidopsis genome, the mutation in which allows to suppress the zip4(s)1 and msh5 mutations associated with disturbances in meiotic division (Crismani et al., 2012). It turned out that the At1g35530 gene encodes a DNA helicase homologous to the human FANCM helicase. In yeast, FANCM orthologues and their cofactors form a conservative complex involved in the formation of non-crossover products during meiosis through disruption of D-loops (Gari et al., 2008). Arabidopsis plants with the mutant fancm gene demonstrate an increase in the frequency of meiotic recombination from 2 to 3.6 times in all eight studied genome regions and are indistinguishable from wild-type plants in terms of growth and fertility (Crismani et al., 2012). Additional crossovers are independent of ZMM proteins and occur via the MUS81 pathway typical to class II (Crismani et al., 2012; Girard et al., 2014).

Thus, it was demonstrated for the first time that FANCM in plants is a strong negative regulator of crossing over. However, subsequent studies showed that the fancm mutation was effective only in arabidopsis inbred lines of the Columbia-0 or Landsberg erecta ecotypes; in hybrids of the Columbia- 0 × Landsberg erecta combination, the fancm mutation does not increase the frequency of meiotic recombination (Girard et al., 2015). In addition, the fancm mutation effectively restores the formation of bivalents in the zmm mutants in the Columbia-0, Landsberg erecta, or Wassilewskija inbred lines, but not in the Columbia-0 × Landsberg erecta and Columbia- 0 × Wassilewskija hybrids.

Later, in arabidopsis plants, the conservative FIDGETINLIKE1 AAA-ATPase (FIGL1) was identified, which also acted as a negative regulator of crossover formation (Girard et al., 2015). It is known that FIGL1 belongs to the FIDGETIN subfamily and is involved in DNA repair (Yuan, Chen, 2013). In arabidopsis, FIGL1, like FANCM (Crismani et al., 2012), limits the formation of crossovers across the entire genome (Girard et al., 2015). In particular, in a single arabidopsis figl1- 1 mutant, the frequency of meiotic recombination increased in each of the six tested sites by an average of 72 % (in single fancm-1 mutants the frequency of meiotic recombination on average tripled (Crismani et al., 2012)) and a noticeable increase in the frequency of recombination took place in the distal regions of chromosomes. A six-fold increase in the frequency of meiotic recombination was found in arabidopsis figl1-1 fancm-1 double mutants compared to wild-type plants in six tested genome regions, while maintaining the progression of meiotic division and fecundity (Girard et al., 2015).

Recent results indicate that the effects of the figl1-1 and fancm-1 mutations are synergistic, thus affecting different metabolic pathways to limit crossing over. It was also found that two figl1 fancm mutations in Columbia-0 × Landsberg erecta hybrids resulted in a 2.5-fold increase in meiotic recombination frequency in the four sites tested compared to wild-type hybrids. This was higher than either of the figl1 or fancm mutants alone (1.8 and 1.2 times, respectively), confirming that figl1 and fancm have a multiplicative effect also in the hybrid genetic environment.

It is assumed that in arabidopsis, the FIGL1 protein negatively affects the dynamics of two conservative recombinases DMC1 and RAD51, counteracting the invasion of singlestranded DNA ends into the homologous chromosome, and thus prevents the interaction between homologous chromosomes (Girard et al., 2015). The available data allow us to conclude that FIGL1 and FANCM represent two sequential barriers against crossing over, the first of which limits the invasion of DNA strands into the homologous chromosome, and the second, due to helicase activity, unwinds intermediate DNA structures that arise during the formation of the D- loop (Girard et al., 2015). This model is supported by direct evidence for physical interaction of the FIGL1 protein via its FRBD domain with RAD51 and DMC1 proteins and an increase in DMC1 foci in arabidopsis figl1 mutants (Fernandes et al., 2018a).

The complex of BTR (BLOOM-TOP3-RMI1-RMI2) in humans and Sgs1-Top3-Rmi1 in budding yeast is highly conservative and plays a major role in the formation of noncrossover products by resolving the double Holliday structure or by disrupting D-loops (Fasching et al., 2015). In particular, during the reaction, two Holliday structures migrate towards each other using the BLOOM/Sgs1 helicase. The structure thus generated is then removed using the TOP3α/Top3 topoisomerase and its cofactors (Berchowitz et al., 2007; Higgins et al., 2008; Macaisne et al., 2011). The same protein complex promotes D-loop unwinding, which results in the formation of exclusively non-crossover products (Crismani et al., 2012; Girard et al., 2014; Mercier et al., 2015).

In arabidopsis, the genome contains three members of the BTR complex: TOP3α and RMI1 as single genes, and the Sgs1 homologue as two paralogous genes, RECQ4A and RECQ4B (Séguéla-Arnaud et al., 2015, 2017). In a recent study, arabidopsis plants carrying different top3α mutant alleles were shown to make a 1.5 to 2.5-fold increase in meiotic recombination frequency. Arabidopsis recq4a recq4b double mutants show a 6.2-fold increase in meiotic recombination frequency compared to wild-type plants (Séguéla-Arnaud et al., 2015). Moreover, the increase in frequency occurred due to the appearance of class II crossovers that are not subject to interference. The effects of the top3α and recq4a recq4b mutations in arabidopsis were enhanced against the background of the fancm mutation. Compared with wild-type plants, the frequency of meiotic recombination increases on average by 4.8 times in the top3α fancm double mutant and by 9 times in the recq4a recq4b fancm triple mutant. These results allowed the authors to state that there are at least two independent pathways for the negative regulation of crossing over in arabidopsis. From the point of view of selective breeding studies, it was important that, despite a significant increase in recombination, the top3α fancm and recq4a recq4b fancm mutants grew normally, were fully fertile and did not show defects in meiotic division (Séguéla-Arnaud et al., 2015, 2017).

The same authors (Fernandes et al., 2018a) showed that when the recq4 mutation and the figl1 mutation are combined in one arabidopsis plant, the frequency of crossing over increases by 7.8 times and the genetic map lengthens from 389 to 3037 cM. It has also been shown that the increase in the number of crossing over events occurs unevenly along the chromosomes and increases from the centromere to the telomere. Finally, female recombination was higher than male recombination in the recq4 figl1 double mutant (3200 versus 2720 cM), although in wild-type plants recombination in male meiosis is much higher than in female meiosis (490 versus 290 cM). These results suggest that the factors that make female meiosis less recombinogenic than male meiosis do not operate in the context of this double mutant.

Almost simultaneously with studies of the role of FANCM in arabidopsis plants, an attempt was made to increase the frequency of meiotic recombination in its close relatives of agricultural importance: diploid turnip plants (Brassica rapa L.) and tetraploid rapeseed plants (B. napus L.) (Blary et al., 2018). In this work, it was found that the braA.fancm-1 missense mutation in the turnip BraA.FANCM gene is able to partially complement the braA.msh4-1 mutation in the meiosis-specific BraA.MSH4 gene of the turnip, which in turn reduces the number of bivalents in metaphase and gives rise to univalents

Turnip double mutants braA.fancm-1 braA.msh4-1 show a 3-fold increase in the number of crossovers, equal to the increase previously observed in arabidopsis (Crismani et al., 2012). In rape plant mutants carrying the bnaA.fancm-1 nonsense mutation in the A genome and the bnaC.fancm-1 or bnaC.fancm-2 missense mutation in the C genome, a certain increase (1.3 times) in the frequency of meiotic recombination was observed. The authors attribute this result to the residual activity of FANCM mutant variants from the C genome in tetraploid rapeseed plants (AACC genome) (Blary et al., 2018).

Also, the influence of FANCM, RECQ4 and FIGL1 factors on the frequency of meiotic recombination was studied in other important agricultural crops: rice, pea (Pisum sativum L.) and cultivated tomato (Mieulet et al., 2018). Mutations in the recq4 orthologue genes increase the frequency of meiotic recombination from 2.7 to 3.7 times in all studied plant species. Mutations in fancm orthologue genes slightly increase the frequency of meiotic recombination, from 1.6 to 2.3 times in pea and rice, but not in tomato, which showed no changes. It was shown that in lettuce (Lactuca sativa L.), knockout of the FANCM gene orthologue using CRISPR/Cas9 genome editing leads to a decrease in the viability of pollen and a decrease in the number of seeds (Li et al., 2021). In lettuce fancm mutants, 78 % of meiocytes in metaphase I have univalents. These results indicate that FANCM in lettuce, in contrast to arabidopsis plants, likely has an additional function in meiosis. Notably, homozygous knockout of figl1 orthologs in tomato, pea, and rice plants induces sterility (Zhang et al., 2017; Mieulet et al., 2018).

Thus, the recq4 mutation increases the frequency of crossing over by about 3 times in all studied crops (rice, pea, and tomato), so manipulation of the RECQ4 gene may be a ver-satile tool to increase meiotic recombination in plants. However, the presented results also indicate that the meiotic effects found in the model object are not always reproduced in agricultural crops.

It has been shown that the frequency of interfering class I crossovers in arabidopsis can be influenced by overexpression of the HEI10 gene (an analogue of Human Enhancer of Invasion 10), which encodes a meiosis-specific E3 ligase associated with quantitative variation in the frequency of crossing over between arabidopsis ecotypes (Ziolkowski et al., 2017). In particular, the frequency of meiotic recombination in transgenic arabidopsis plants of both Columbia-0 and Landsberg erecta ecotypes or their hybrid Columbia-0 × Landsberg erecta significantly increases and shows a positive correla- tion with the expression level of the HEI10 transgene. The population of transgenic plants based on the Columbia-0 ecotype with overexpression of the HEI10 gene contained more than twice as many crossovers, which was revealed using the MLH1 protein, a marker for class I crossovers. A simultaneous increase in the number of copies of the HEI10 gene and knockout of the RECQ4A and RECQ4B genes in arabidopsis lead to a 5-fold increase in meiotic recombination in chromosome arms and to a 1.5-fold increase in pericentromeric heterochromatin (Serra et al., 2018). Thus, the combination of overexpression of the HEI10 gene with suppression of the expression of the RECQ4A and RECQ4B genes for the first time made it possible to simultaneously increase the number of class I and II crossovers

## Conclusion

Over the past two decades, numerous studies have been performed that allowed to reveal key elements of the control of meiotic crossing over, which can be used to increase the frequency of crossover exchanges and redistribute their positions along the chromosomes. The experiments on overexpression of the HEI10 crossover enhancer gene and inactivation of the FANCM, RECQ4, and FIGL1 crossover repressor genes in arabidopsis plants turned out to be the most promising. Combining these experimental approaches has significantly increased the frequency and distribution of class I and II crossovers. The results obtained in arabidopsis opened up the possibility of manipulating the process of meiotic recombination in agricultural plant species. However, the results obtained on the model object are not always reproducible on agricultural crops. Obviously, additional efforts are needed to reveal the features of the functioning of orthologues of these genes in various plant genomes.

## Conflict of interest

The authors declare no conflict of interest.
